# Construction of Bi_2_MoO_6_/Ag_2_CrO_4_ Heterojunction Nanocomposites with Enhanced Visible-Light Photocatalytic Activity and Mechanistic Insight

**DOI:** 10.3390/nano16171079

**Published:** 2026-08-30

**Authors:** Weijie Hua, Songhua Huang, Huixin Yuan

**Affiliations:** School of Intelligent Equipment Engineering, Wuxi Taihu University, Wuxi 214064, China

**Keywords:** Bi_2_MoO_6_/Ag_2_CrO_4_, n–n heterojunction, visible-light photocatalysis, Rhodamine B, charge-transfer mechanism

## Abstract

In this work, flower-like Bi_2_MoO_6_ microspheres were first prepared by solvothermal synthesis. Ag_2_CrO_4_ nanoparticles were then deposited in situ onto the Bi_2_MoO_6_ surface, leading to the formation of a Bi_2_MoO_6_/Ag_2_CrO_4_ n–n heterojunction nanocomposite photocatalyst. Through characterization technologies and visible-light degradation experiments, the photocatalytic behavior and degradation mechanisms of nanocomposites were explored. The results demonstrated that Ag_2_CrO_4_ block-like particles were uniformly anchored onto the surface of the flower-like Bi_2_MoO_6_ microspheres. The diffraction peaks, lattice fringes, XPS binding energies and FT-IR absorption bands of the composite samples were in good agreement with those of the individual components. Construction of the heterojunction remarkably broadened the optical response of Bi_2_MoO_6_, extending the absorption edge from 497 to 713 nm. The band gap decreased to 1.52 eV, lower than the values measured for either constituent semiconductor. Moreover, the nanocomposite showed a markedly lower photoluminescence emission intensity. The Bi_2_MoO_6_/Ag_2_CrO_4_ photocatalyst removed maximum 99.43% of Rhodamine B (RhB) within 60 min under visible-light illumination. The photocatalytic activity of nanocomposite with a Bi_2_MoO_6_:Ag_2_CrO_4_ molar ratio of 1:1 was 3.56 times that of pure Bi_2_MoO_6_, while the kinetic constant reached 0.0570 min^−1^, exceeding those of Bi_2_MoO_6_ and Ag_2_CrO_4_ by factors of 13.90 and 2.26, respectively. After five consecutive reuse cycles, the photocatalyst still removed more than 85% of RhB from aqueous solution. Optimal degradation performance was obtained using 0.50 g/L of photocatalyst and the initial RhB concentration of 10 mg/L. The photocatalytically generated active species h^+^ and ·O_2_^−^ can effectively decompose the chromophores of RhB molecules in water. Finally, the degradation mechanism of RhB by nanocomposites was proposed.

## 1. Introduction

Wastewater containing synthetic dyes is widely produced during textile dyeing, leather finishing, papermaking and various chemical manufacturing processes. Such effluents usually contain high concentrations of persistent organic contaminants and exhibit strong coloration, complex compositions, and considerable toxicity, thereby presenting serious environmental concerns [1]. Global expansion of textile manufacturing has resulted in a substantial increase in the production of dye-laden wastewater. It has been reported that approximately 150–200 L of water is consumed to produce 1 kg of textiles during the dyeing and finishing process, while the wastewater generated during this stage accounts for approximately 70–80% of the total wastewater discharged by the textile industry [2]. Due to the limited dye fixation efficiency, approximately 10–15% of the applied dyes fail to bind to textile fibers during the dyeing process and are directly discharged into the environment with the wastewater [2]. The discharge of large volumes of untreated or inadequately treated dye wastewater into natural water bodies significantly increases water color intensity, reduces light penetration, inhibits the photosynthesis of algae and aquatic plants, decreases dissolved oxygen levels, and ultimately disrupts the stability of aquatic ecosystems. Moreover, azo dyes, anthraquinone dyes and their degradation products present in dye wastewater exhibit considerable toxicity. Some dye-related pollutants are characterized by mutagenicity, carcinogenicity and a strong tendency to bioaccumulate. Once introduced into food webs, they may progressively accumulate and threaten ecological integrity as well as public health [3]. Rhodamine B exhibits inhibitory effects on aquatic organisms, such as significant growth suppression and oxidative stress responses in the freshwater microalga chlorella vulgaris [4]. Driven by the pursuit of carbon neutrality and the principles of the circular economy, semiconductor photocatalytic technology has attracted widespread attention because of its environmental friendliness, low energy consumption and ability to utilize solar energy effectively [5]. Heterogeneous photocatalysis can directly drive the degradation of organic pollutants under ambient temperature and pressure using solar light, offering advantages such as easy catalyst separation, reusability, and a green, sustainable process. Semiconductor photocatalysis is of great theoretical significance and practical value for safeguarding aquatic ecosystems, promoting wastewater resource recovery and advancing sustainable industrial development. The photocatalytic performance of conventional semiconductor materials remains far from satisfactory because only a limited fraction of the solar spectrum can be effectively utilized, while photoexcited charge carriers readily undergo recombination before participating in surface reactions. In addition, sluggish charge transport across interfaces further reduces the utilization efficiency of photogenerated carriers [6]. Accordingly, developing semiconductor photocatalysts with enhanced visible-light utilization and efficient charge-separation capability has become an important objective in current photocatalysis research.

Benefiting from their favorable band structures, plasmon-enhanced light absorption and abundant surface-active sites, silver-based semiconductor nanomaterials have demonstrated considerable potential for visible-light-driven photocatalytic processes [7]. Owing to its band gap of about 1.8 eV, silver chromate (Ag_2_CrO_4_) can effectively harvest visible light. The valence- and conduction-band edge potentials are approximately 2.24 and 0.49 eV, respectively, enabling the generated holes to participate in strong oxidation reactions [8]. The crystal structure of Ag_2_CrO_4_ consists of AgO_4_ tetrahedra, CrO_4_ tetrahedra and AgO_6_ octahedra [9]. Its characteristic brick-red color reflects its excellent visible-light absorption capability, suggesting that Ag_2_CrO_4_ is a promising visible-light-responsive photocatalyst. Nevertheless, several issues remain to be further investigated. For instance, the photocorrosion of Ag_2_CrO_4_ and effective strategies for enhancing its photocatalytic performance are still major challenges. Luo et al. [10] coupled Ag_2_CrO_4_ with the narrow-band-gap semiconductor In_2_S_3_ to construct an In_2_S_3_/Ag_2_CrO_4_ heterostructure, in which the charge-transfer process followed an all-solid-state Z-scheme mechanism. Pirhashemi et al. [11] fabricated the ZnO/Ag_2_CrO_4_ heterojunction, and the resulting photocatalyst exhibited enhanced photocatalytic performance toward the degradation of organic dyes. Shaker et al. [12] synthesized the ternary ZnO/AgI/Ag_2_CrO_4_ photocatalyst, whose enhanced photocatalytic performance was attributed to the broadened light-response range and the accelerated migration of photogenerated charge carriers.

Bismuth-based semiconductors exhibit favorable electronic structures together with efficient light absorption, making them attractive materials for photocatalytic applications [13]. Bismuth molybdate is a visible-light-driven photocatalyst that has received extensive attention in recent years because of its low toxicity, high photocatalytic efficiency and excellent visible-light responsiveness [14,15]. γ-Bi_2_MoO_6_ is a typical Aurivillius-type ternary oxide. Its crystal structure belongs to the layered perovskite family and is composed of alternating perovskite-like (MoO_4_)^2+^ layers and fluorite-like (Bi_2_O_2_)^2+^ layers [16]. The optical absorption edge of γ-Bi_2_MoO_6_ reaches nearly 490 nm because of its moderate band gap (2.6–2.8 eV) [17]. Furthermore, the periodic stacking of atomic layers facilitates directional migration of photoexcited charge carriers, improving their separation efficiency [18,19]. Although Bi_2_MoO_6_ exhibits favorable photocatalytic properties, its practical performance is restricted by inefficient harvesting of visible light and fast recombination of charge carriers generated during photoexcitation [20]. As a semiconductor with a tunable electronic band structure, Bi_2_MoO_6_ can be coupled with other semiconductors to construct heterojunction photocatalysts. Feng et al. [21] synthesized the CdS/Bi_2_MoO_6_ composite with a type-II heterojunction structure via the hydrothermal method. The CS/BMO-1 composite achieved degradation efficiencies of 100% and 92% toward Rhodamine B and tetracycline, respectively, under visible-light irradiation. Chao et al. [22] reported the fabrication of Z-scheme Bi_4_O_5_I_2_/Bi_2_MoO_6_ nanocomposite using a solvothermal approach. Photocatalytic evaluation revealed that the BIB-2 sample exhibited the most efficient degradation of tetracycline, with 91.8% of the pollutant eliminated after 60 min of visible-light exposure. Yao et al. [23] reported the fabrication of an internal electric field-assisted S-scheme CdIn_2_S_4_/Bi_2_MoO_6_ heterojunction using a hydrothermal approach. The resulting 25–CIS/BMO microspheres exhibited outstanding activity toward levofloxacin degradation, eliminating 93.7% of the pollutants after 90 min of illumination.

To overcome the inherent drawbacks of single-component photocatalysts, an n–n Bi_2_MoO_6_/Ag_2_CrO_4_ heterojunction was designed by depositing Ag_2_CrO_4_ onto Bi_2_MoO_6_ through an in situ precipitation strategy. This modification restrains the photocorrosion behavior of Ag_2_CrO_4_ and meanwhile extends the spectral absorption region of Bi_2_MoO_6_. The resulting composite was applied to the visible-light degradation of Rhodamine B (RhB, typical cationic organic dye) in water. Its crystal structure, morphology, optical response and physicochemical properties were comprehensively analyzed using XRD, SEM, TEM, XPS, FT-IR, UV-Vis DRS and PL to establish the relationship between material properties and photocatalytic behavior. The photocatalytic activity of the heterojunction was further assessed through RhB degradation experiments, with particular attention given to its reusability, operational stability and charge-transfer mechanism. The influences of catalyst dosage and initial pollutant concentration on the degradation process were also examined. In addition, active-species quenching experiments together with the evolution of RhB UV–Vis absorption spectra were employed to clarify the degradation mechanism and identify the predominant active components involved in the photocatalytic reaction. The formation of the Bi_2_MoO_6_/Ag_2_CrO_4_ heterojunction effectively alleviated the photocorrosion of Ag_2_CrO_4_ while extending the visible-light response of Bi_2_MoO_6_. These characteristics highlight the potential of the Bi_2_MoO_6_/Ag_2_CrO_4_ heterojunction as an efficient visible-light photocatalyst for the removal of refractory organic contaminants from wastewater.

## 2. Materials and Methods

### 2.1. Chemicals

Bismuth nitrate pentahydrate (Bi(NO_3_)_3_·5H_2_O, 99%), sodium molybdate dihydrate (Na_2_MoO_4_·2H_2_O, 99%), ethylene glycol, potassium chromate (K_2_CrO_4_, 99.5%), Rhodamine B (RhB), tert-butanol (TBA), triethanolamine (TEOA) and *p*-benzoquinone (BQ) were obtained from Macklin Biochemical Co., Ltd. (Shanghai, China). Absolute ethanol and silver nitrate (AgNO_3_) were supplied by Meryer Chemical Technology Co., Ltd. (Shanghai, China) and Xijing Biotechnology Co., Ltd. (Fuzhou, China), respectively. Unless otherwise specified, commercially available analytical-grade chemicals were used as received. Deionized water (electrical conductivity: 1 μS/cm) was utilized for solution preparation and all subsequent experimental procedures.

### 2.2. Synthesis of Bi_2_MoO_6_

Flower-like microspherical Bi_2_MoO_6_ was successfully synthesized via a solvothermal method using Bi(NO_3_)_3_·5H_2_O and Na_2_MoO_4_·2H_2_O as the Bi and Mo precursors, respectively. First, 1.6866 g of Bi(NO_3_)_3_·5H_2_O and 0.4210 g of Na_2_MoO_4_·2H_2_O were separately dissolved in 5 mL of ethylene glycol under ultrasonic treatment until clear solutions were obtained. Subsequently, the two solutions were slowly mixed under continuous magnetic stirring. After thorough mixing, 20 mL of absolute ethanol was added dropwise to the mixed solution, followed by vigorous stirring for 30 min until the homogeneous transparent solution was formed. The precursor dispersion was introduced into a 100 mL Teflon-lined stainless-steel vessel for solvothermal treatment at 160 °C for 24 h. After the autoclave had cooled to room temperature, the precipitate was isolated by filtration, successively washed with deionized water and anhydrous ethanol, and subsequently dried at 60 °C for 12 h. The obtained solid was first dried, then cooled to room temperature under natural conditions and milled into a fine powder, resulting in pale-yellow Bi_2_MoO_6_.

### 2.3. Synthesis of Bi_2_MoO_6_/Ag_2_CrO_4_

Bi_2_MoO_6_/Ag_2_CrO_4_ heterojunction composites were synthesized via an in situ chemical deposition method. Four composite samples with Bi_2_MoO_6_-to-Ag_2_CrO_4_ molar ratios of 0.5:1, 1:1, 2:1 and 4:1 were prepared and denoted as BMAC0.5, BMAC1, BMAC2 and BMAC4, respectively. The preparation procedure of the BMAC1 sample is described as a representative example. Bi_2_MoO_6_ (0.2 mmol) was introduced into 40 mL of deionized water and subjected to 30 min of sonication to produce a well dispersed suspension, designated as Solution A. Separately, K_2_CrO_4_ (0.2 mmol) was completely dissolved in 15 mL of deionized water after 5 min of ultrasonic treatment, and the resulting solution was labeled as Solution B. Under continuous magnetic stirring, 4 mL of 0.1 M AgNO_3_ solution was added dropwise to Solution A and the suspension was further stirred for 30 min to ensure the uniform adsorption of Ag^+^ onto the surface of Bi_2_MoO_6_. Thereafter, Solution B was slowly added dropwise to the suspension under vigorous stirring, and the reaction was allowed to proceed for 2 h. During this process, the color of the suspension gradually changed from pale yellow to dark red. Following filtration, the precipitate was thoroughly washed with deionized water and anhydrous ethanol, then dried at 60 °C for 12 h. The dried solid was then milled into fine powder to produce the dark-red Bi_2_MoO_6_/Ag_2_CrO_4_ photocatalyst. Pure Ag_2_CrO_4_ was obtained by repeating the same synthetic procedure while omitting the Bi_2_MoO_6_.

### 2.4. Characterization

The phase compositions of the synthesized photocatalysts were identified by X-ray diffraction (XRD, SmartLab SE, Rigaku, Tokyo, Japan) using Cu Kα radiation. Surface morphology together with elemental distribution was analyzed using field-emission scanning electron microscopy coupled with energy-dispersive X-ray spectroscopy (SEM, Sigma 360, ZEISS, Oberkochen, Germany; EDS, Xplore 30, Oxford Instruments, Abingdon, UK). The microstructure and lattice characteristics were further examined by transmission electron microscopy (TEM, JEM-F200, JEOL, Yokohama, Japan). Fourier transform infrared spectroscopy (FT-IR, Nicolet iS20, Thermo Fisher Scientific, Waltham, MA, USA) was employed to investigate the chemical bonding environment, while X-ray photoelectron spectroscopy (XPS, K-Alpha, Thermo Scientific, Waltham, MA, USA) was used to determine the elemental chemical states and valence-band information. Optical absorption properties were evaluated from UV–Vis diffuse reflectance spectra (UV-Vis DRS) recorded on a UV-3600i Plus spectrophotometer (Shimadzu, Kyoto, Japan) with BaSO_4_ as the reflectance standard. An FLS1000 photoluminescence spectrometer (PL, Edinburgh Instruments, Abingdon, UK) was employed to acquire PL spectra under 370 nm excitation, providing information on the recombination behavior of photoinduced charge carriers.

### 2.5. Evaluation of Photocatalytic Activity

To evaluate the photocatalytic performance of Bi_2_MoO_6_, Ag_2_CrO_4_ and Bi_2_MoO_6_/Ag_2_CrO_4_ nanocomposites, Rhodamine B (RhB) aqueous solution was selected as the target contaminant for photocatalytic degradation. A 300 W xenon lamp (YM-GHX-XE-300, YuMing, Shanghai, China) fitted with the 400 nm cutoff filter served as the visible-light source. Photocatalytic reactions were performed in a water-cooled double-layer quartz reactor, where circulating water continuously regulated the reaction temperature close to ambient conditions. For each experiment, 50 mg of catalyst was introduced into 100 mL of a 10 mg/L RhB solution, followed by magnetic stirring in the dark for 30 min to reach adsorption equilibrium. Before light exposure, a 4 mL suspension was withdrawn as the initial sample. During irradiation, additional 4 mL aliquots were collected every 10 min and centrifuged at 10,000 r/min to remove the catalyst particles. The concentration of RhB was determined from the absorbance of the supernatant at 554 nm using the UV–Vis spectrophotometer (L8, YOKE, Shanghai, China). In the free radical scavenging experiments, TBA, TEOA and BQ were used as scavengers for ·OH, h^+^ and ·O_2_^−^, respectively. The respective scavengers were added before the photoreaction began. The stability experiment procedure was as follows: After a single photocatalytic degradation reaction of RhB was completed, the suspension was filtered and washed three times with deionized water and anhydrous ethanol, and the sample was collected and dried at 60 °C for 12 h. The dried samples were then used in the next photodegradation experiment under the same experimental conditions. The degradation efficiency together with the apparent reaction rate constant was subsequently calculated using Equations (1) and (2).

(1) $$\eta =\frac{C_{0}-C_{t}}{C_{0}}$$

(2) $$\ln(\frac{C_{0}}{C_{t}})=kt$$

In Equations (1) and (2), *η* refers to the percentage degradation of RhB; *C*_0_ and *C_t_* indicate the initial and time-dependent RhB concentrations (mg/L), respectively; *k* represents the kinetic coefficient (min^−1^), and *t* corresponds to the irradiation period (min).

## 3. Results and Discussion

### 3.1. XRD Characterization

**Figure 1:**
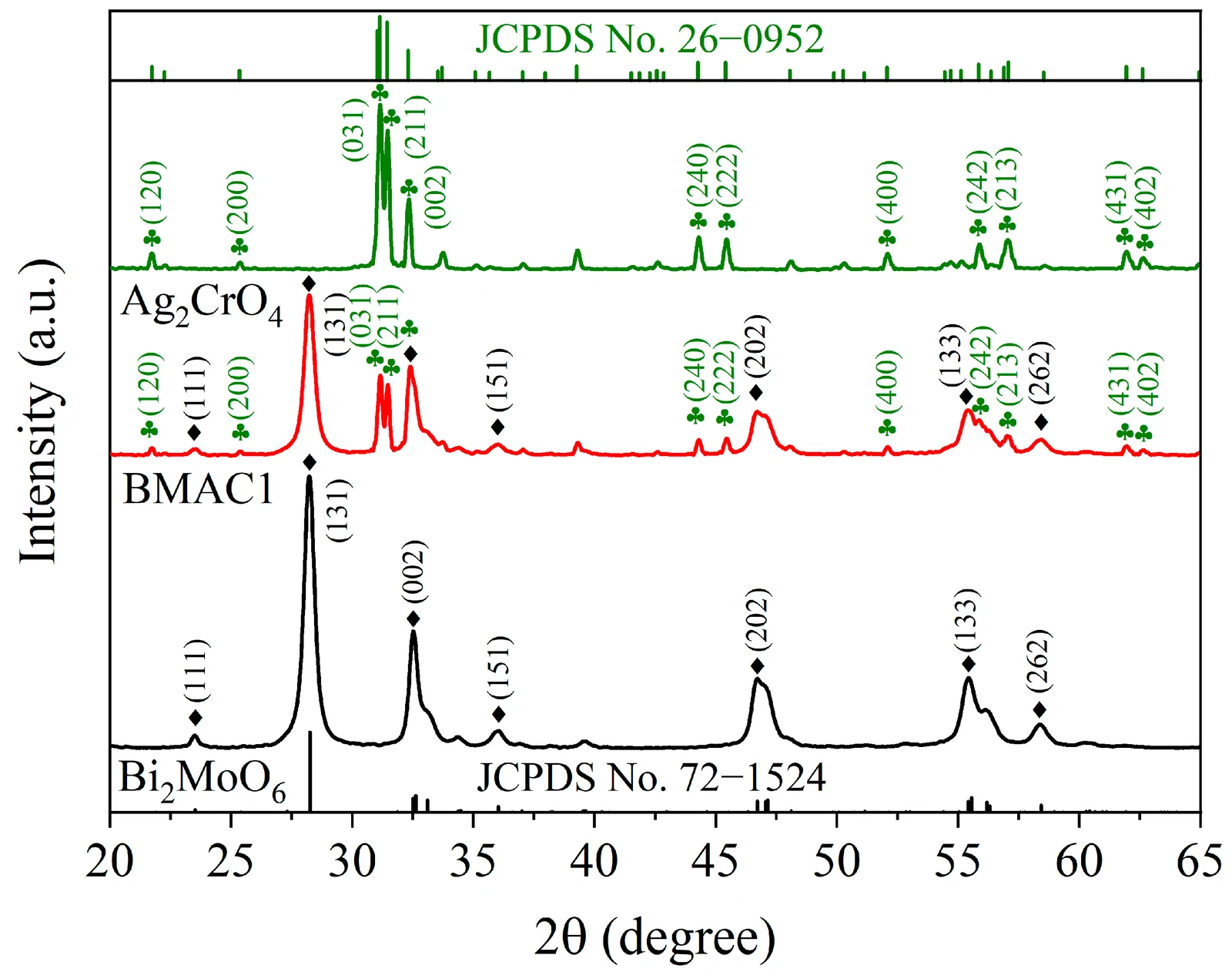
XRD patterns of Bi_2_MoO_6_, Ag_2_CrO_4_ and BMAC1.

Figure 1 presents the XRD patterns of pristine Bi_2_MoO_6_, pristine Ag_2_CrO_4_ and the BMAC1 composite. Pristine Bi_2_MoO_6_ displays a series of diffraction peaks at 23.50°, 28.22°, 32.52°, 36.06°, 46.72°, 55.42° and 58.38°. These peaks can be attributed to the (111), (131), (002), (151), (202), (133) and (262) crystal planes of orthorhombic Bi_2_MoO_6_, in accordance with JCPDS card no. 72-1524. The diffraction pattern of Ag_2_CrO_4_ agrees well with the standard card (JCPDS no. 26-0952). The characteristic diffraction peaks located at 21.72°, 25.38°, 31.16°, 31.46°, 32.34°, 44.30°, 45.44°, 52.10°, 55.88°, 57.04°, 61.94° and 62.64° can be indexed to the (120), (200), (031), (211), (002), (240), (222), (400), (242), (213), (431) and (402) crystal planes of orthorhombic Ag_2_CrO_4_, respectively. The simultaneous appearance of the diffraction reflections assigned to Bi_2_MoO_6_ and Ag_2_CrO_4_ in the BMAC1 sample verifies that both phases were successfully integrated into the composite heterostructure. Moreover, no noticeable shift in the diffraction peak positions or impurity peaks was observed, indicating that the Bi_2_MoO_6_/Ag_2_CrO_4_ heterojunction was successfully synthesized with high crystallinity.

### 3.2. Microstructural Analysis

#### 3.2.1. Morphological and Elemental Characterization

**Figure 2:**
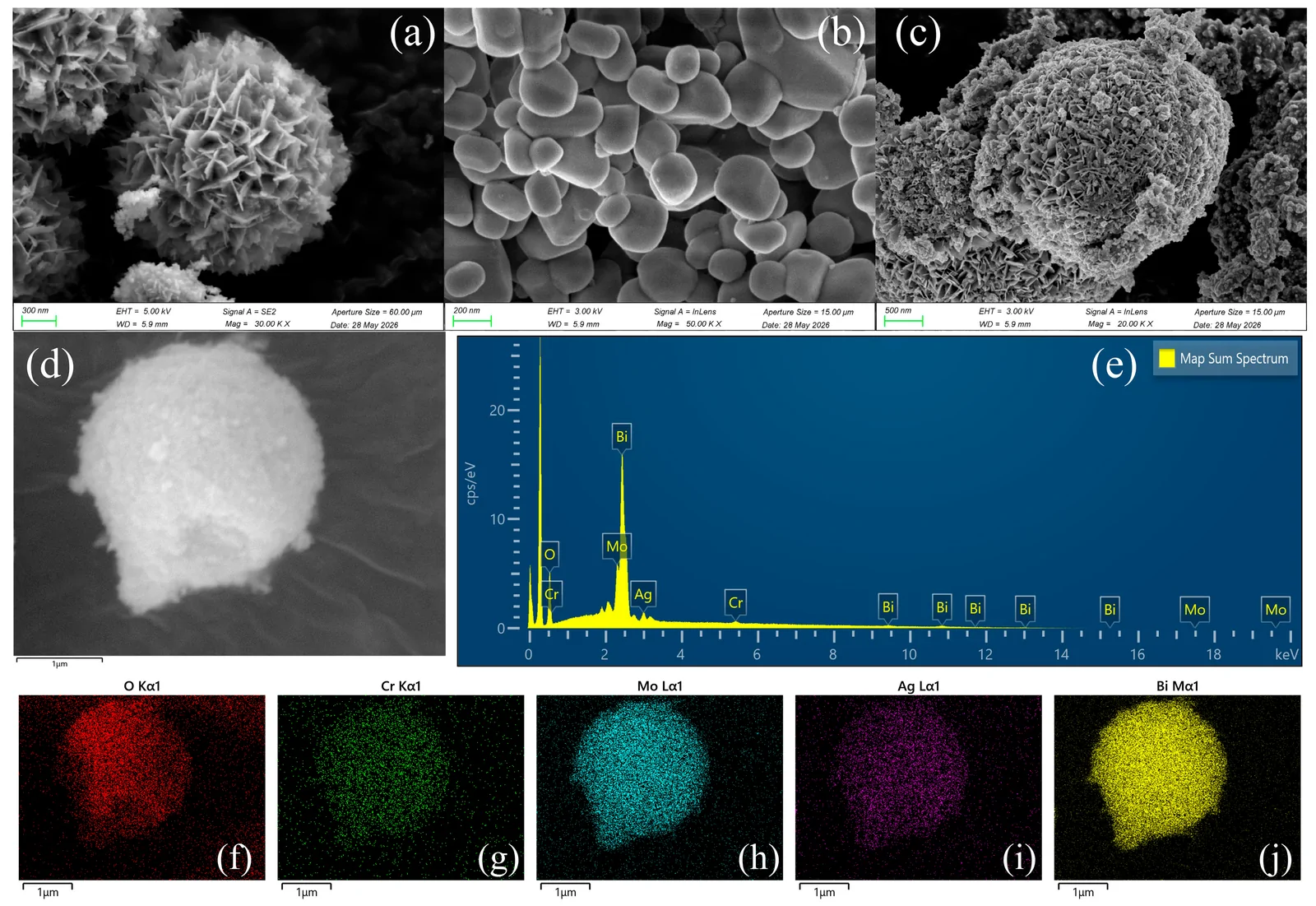
SEM characterization of Bi_2_MoO_6_ (**a**), Ag_2_CrO_4_ (**b**) and BMAC1 (**c**), along with the corresponding EDS spectra (**d**,**e**) and EDS mapping results of BMAC1 (**f**–**j**).

The surface morphologies of Bi_2_MoO_6_, Ag_2_CrO_4_ and BMAC1 were characterized by SEM. Figure 2 summarizes the characterization results. According to Figure 2a, as-prepared Bi_2_MoO_6_ exhibited a uniform flower-like microspherical morphology assembled from numerous interconnected nanosheets. The diameter of an individual flower-like microsphere was approximately 2 μm. Figure 2b shows that the synthesized Ag_2_CrO_4_ consisted of irregular block-like particles with different sizes, which were uniformly distributed despite a certain degree of aggregation. After the successful combination of Bi_2_MoO_6_ and Ag_2_CrO_4_, the block-like Ag_2_CrO_4_ particles were uniformly anchored onto the surface of the flower-like Bi_2_MoO_6_ microspheres, as shown in Figure 2c. In addition, some fine Ag_2_CrO_4_ nanoparticles were observed around the Bi_2_MoO_6_ nanosheets, resulting in an increased specific surface area. The formation of the heterojunction increased the interfacial contact area between Bi_2_MoO_6_ and Ag_2_CrO_4_, thereby providing more active sites for the photocatalytic reaction. The composite sample simultaneously retained the characteristic morphological features of both Bi_2_MoO_6_ and Ag_2_CrO_4_. The EDS spectra confirmed that the BMAC1 heterojunction photocatalyst was composed of Bi, Mo, O, Cr and Ag elements (Figure 2d,e). The elemental mapping images (Figure 2f–j) further revealed that O, Cr, Mo, Ag and Bi were uniformly distributed over the surface of the microspheres, with stronger signals for Bi, Mo and O, which are consistent with the theoretical composition. The morphology and elemental distribution analyses indicate that Ag_2_CrO_4_ block-like crystallites are well dispersed across the surface of Bi_2_MoO_6_ microspheres.

#### 3.2.2. TEM Analysis

**Figure 3:**
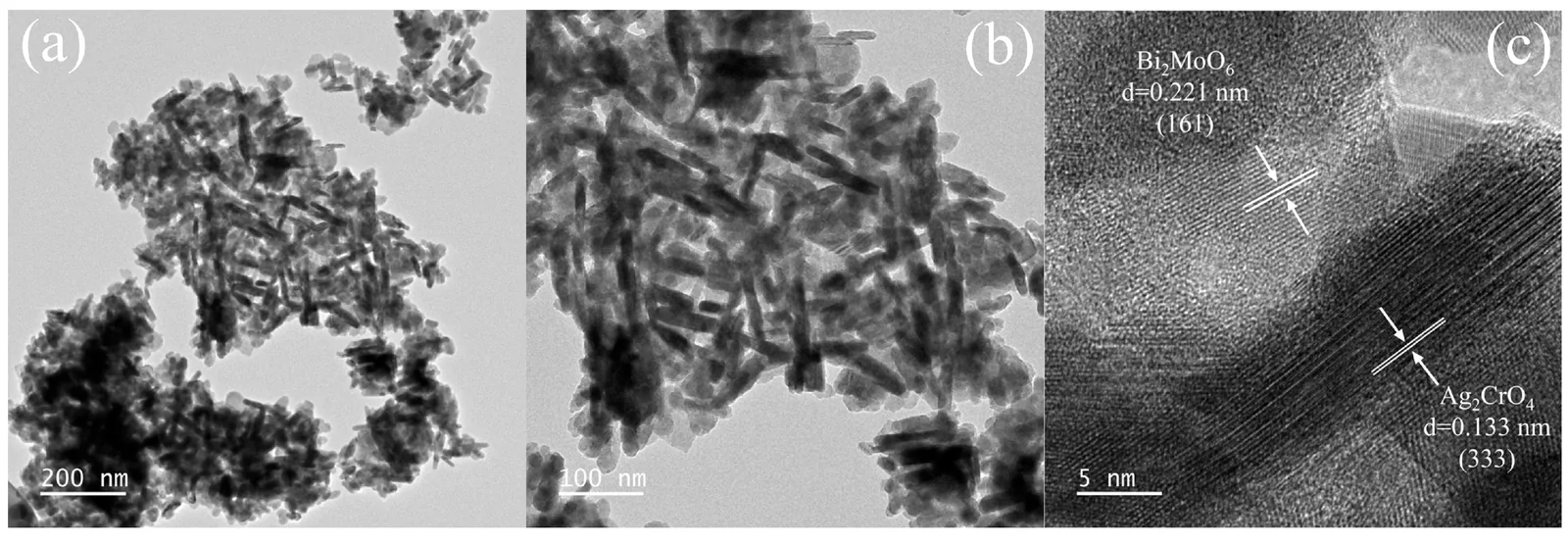
TEM micrographs (**a**,**b**) and HRTEM micrograph (**c**) of BMAC1.

The microstructure of the BMAC1 composite was further investigated by TEM, and the results are shown in Figure 3. As shown in Figure 3a,b, the Ag_2_CrO_4_ nanoparticles were uniformly grown and anchored onto the surface of the Bi_2_MoO_6_ microspheres. An intimate interface is observed between Bi_2_MoO_6_ and Ag_2_CrO_4_, which favors charge transfer across the heterojunction. High-resolution TEM analysis clearly distinguishes the two semiconductor phases within the composite (Figure 3c). The lattice spacing measured from the Ag_2_CrO_4_ region is 0.133 nm, matching the (333) plane, while a spacing of 0.221 nm is identified in the Bi_2_MoO_6_ region and is attributed to the (161) plane. The agreement between the HRTEM measurements and XRD results confirms the coexistence of both crystalline phases and verifies the successful fabrication of the Bi_2_MoO_6_/Ag_2_CrO_4_ heterostructure.

### 3.3. XPS Analysis

**Figure 4:**
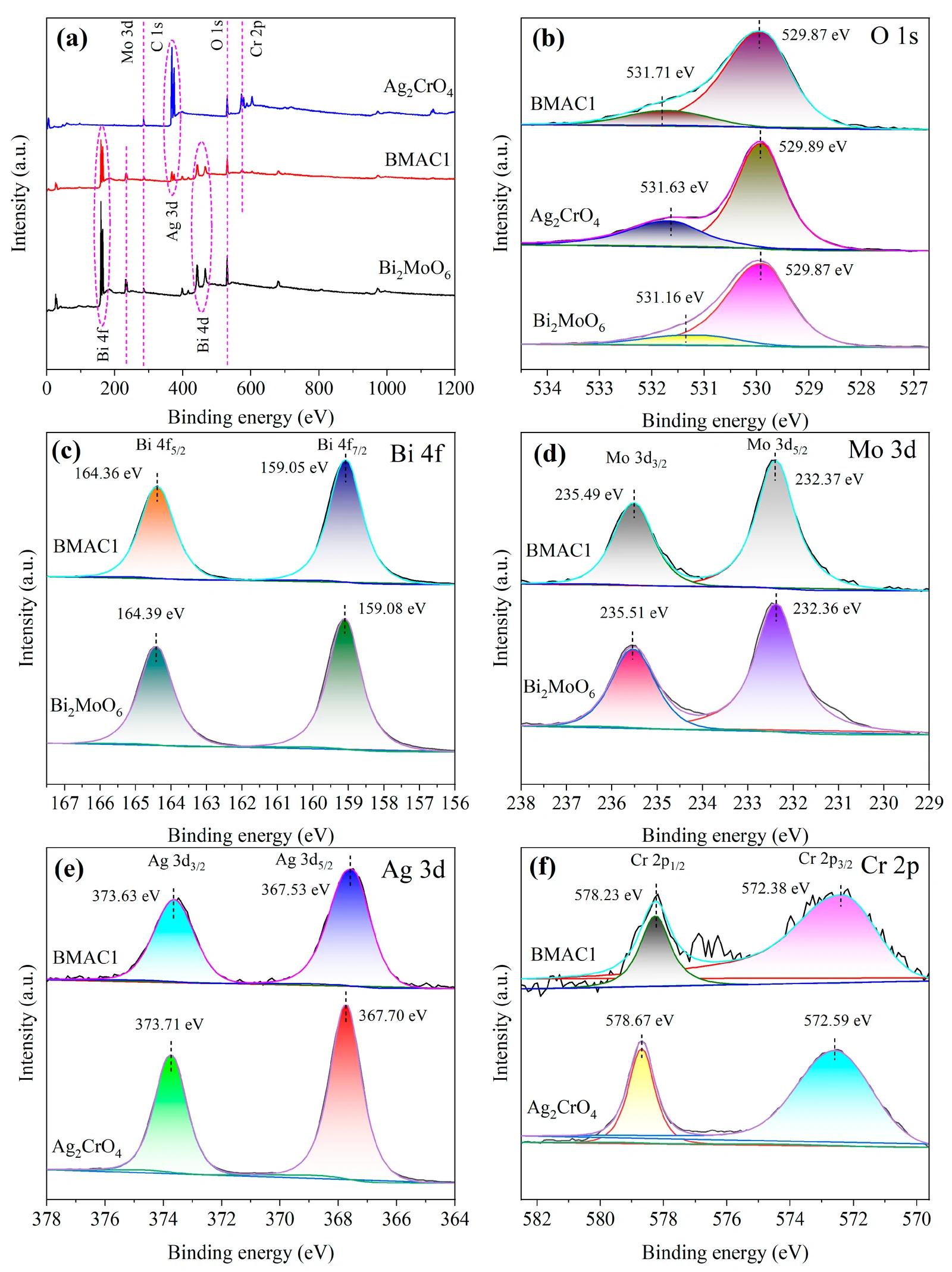
XPS results for Bi_2_MoO_6_, Ag_2_CrO_4_ and BMAC1, including the survey spectrum (**a**) and the corresponding high-resolution spectra of O 1s (**b**), Bi 4f (**c**), Mo 3d (**d**), Ag 3d (**e**) and Cr 2p (**f**).

The surface chemical environments and binding-energy distributions of Bi_2_MoO_6_, Ag_2_CrO_4_ and BMAC1 were further characterized by XPS. The energy scale of the XPS measurements was corrected by setting the C 1s peak to 284.80 eV. The survey and high-resolution spectra are summarized in Figure 4. As shown in Figure 4a, the survey spectrum of Bi_2_MoO_6_ exhibits the characteristic photoelectron peaks of Bi, Mo and O, whereas that of Ag_2_CrO_4_ contains the characteristic peaks of Cr, Ag and O. More importantly, all of these characteristic photoelectron peaks are simultaneously observed in the survey spectrum of the BMAC1 composite. Figure 4b–f present the high-resolution XPS spectra of the O 1s, Bi 4f, Mo 3d, Ag 3d and Cr 2p orbitals. As shown in Figure 4b, the fitted peaks located at 529.87, 529.89 and 529.87 eV are assigned to lattice oxygen, whereas those located at 531.16, 531.63 and 531.71 eV are attributed to surface hydroxyl oxygen species [24]. The high-resolution Bi 4f and Mo 3d spectra of Bi_2_MoO_6_ and BMAC1 are presented in Figure 4c,d. For the Bi_2_MoO_6_ sample, the two characteristic peaks located at 159.08 and 164.39 eV correspond to the Bi 4f_7/2_ and Bi 4f_5/2_ orbitals, respectively, while the corresponding peaks of BMAC1 slightly shift to 159.05 and 164.36 eV, respectively [25]. For pristine Bi_2_MoO_6_, the Mo 3d spectrum is composed of two characteristic signals located at 232.36 and 235.51 eV, which are attributed to the Mo 3d_5/2_ and Mo 3d_3/2_ orbitals. In comparison, the BMAC1 composite displays these signals at 232.37 and 235.49 eV, respectively. The Bi 4f and Mo 3d spectra confirm the presence of Bi^3+^ and Mo^6+^ species [26]. The shifts in the binding energies of the Bi and Mo core levels after composite formation indicate enhanced chemical interactions between Bi_2_MoO_6_ and Ag_2_CrO_4_. The high-resolution Ag 3d and Cr 2p spectra of Ag_2_CrO_4_ and BMAC1 are shown in Figure 4e,f. In the XPS spectrum of pristine Ag_2_CrO_4_, the Ag element is characterized by two binding-energy peaks at 367.70 and 373.71 eV, corresponding to the Ag 3d_5/2_ and Ag 3d_3/2_ levels. Meanwhile, the Cr 2p spectrum contains two components at 572.59 and 578.67 eV, which are characteristic of Cr^6+^ species [27]. For the BMAC1 composite, the binding energies of the Ag 3d_5/2_ and Ag 3d_3/2_ orbitals shift to 367.53 and 373.63 eV, respectively, while those of the Cr 2p_3/2_ and Cr 2p_1/2_ orbitals shift to 572.38 and 578.23 eV, respectively. These results could confirm the existence of Ag^1+^ and Cr^6+^ species. Moreover, the binding energies of all the constituent elements exhibit different degrees of shift compared with those of the corresponding pristine components, further demonstrating the existence of strong interfacial chemical interactions between Bi_2_MoO_6_ and Ag_2_CrO_4_ and confirming the successful formation of the heterojunction.

### 3.4. FT-IR Analysis

**Figure 5:**
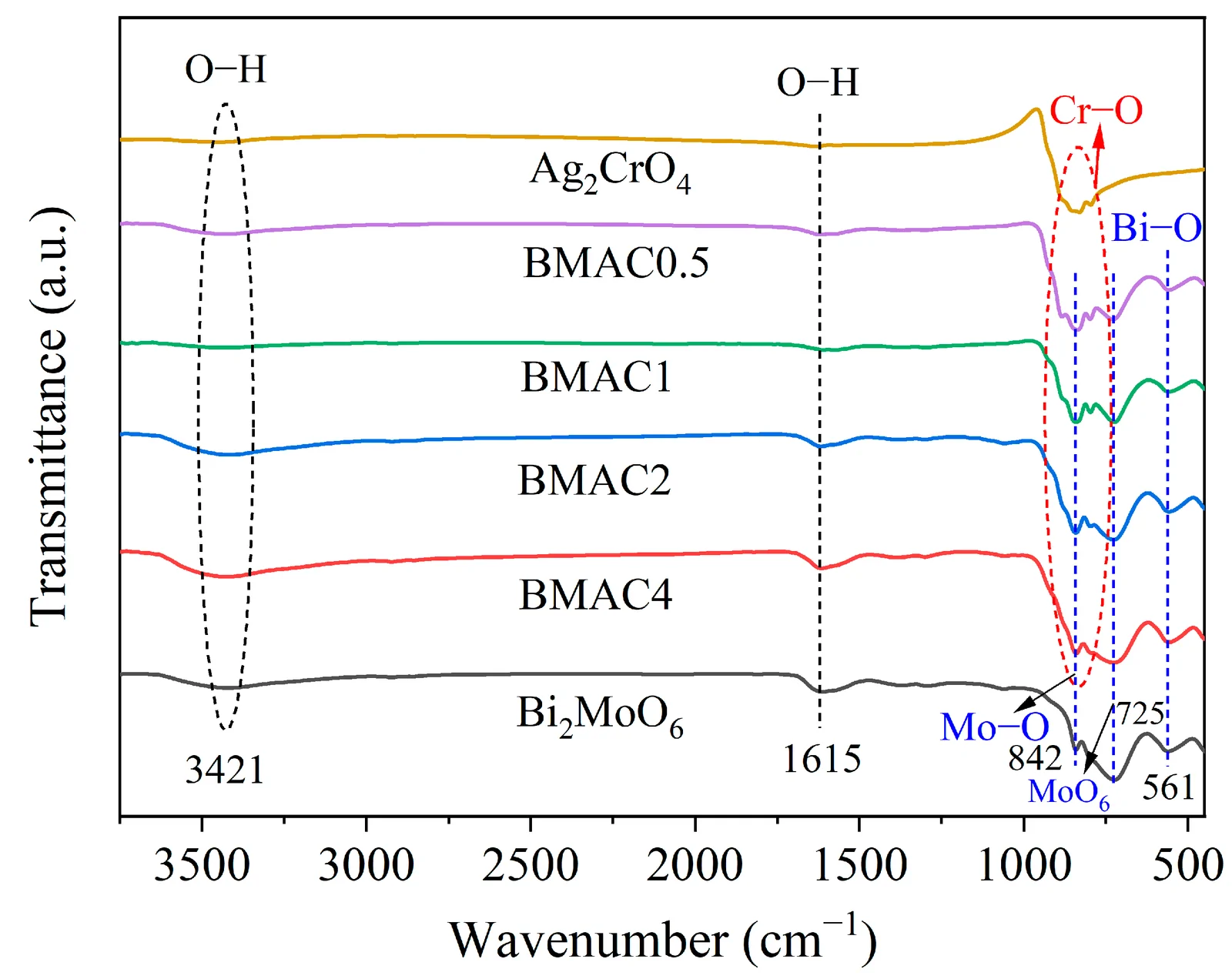
FT-IR spectra of Bi_2_MoO_6_, Ag_2_CrO_4_ and the Bi_2_MoO_6_/Ag_2_CrO_4_ composite.

The functional groups of Bi_2_MoO_6_, Ag_2_CrO_4_ and the Bi_2_MoO_6_/Ag_2_CrO_4_ composite were characterized by FT-IR spectroscopy, and the corresponding spectra are presented in Figure 5. As shown in Figure 5, the FT-IR spectrum of the Bi_2_MoO_6_/Ag_2_CrO_4_ composite contains all the characteristic absorption bands of the individual components (Bi_2_MoO_6_ and Ag_2_CrO_4_). For both Bi_2_MoO_6_ and Bi_2_MoO_6_/Ag_2_CrO_4_, the absorption band centered at 561 cm^−1^ is assigned to the stretching vibration of the Bi–O bond [28]. The absorption band located at 725 cm^−1^ is attributed to the asymmetric stretching vibration of MoO_6_ octahedra. The absorption band at 842 cm^−1^ originates from the stretching vibration of the Mo–O bond [29]. For Ag_2_CrO_4_ and Bi_2_MoO_6_/Ag_2_CrO_4_, the broad absorption band in the range of 831–842 cm^−1^ is assigned to the stretching vibration of the Cr–O bond [30]. With increasing Ag_2_CrO_4_ loading, the intensity of this characteristic absorption band gradually increased. The FT-IR spectra of all samples display two broad absorption peaks near 1615 and 3421 cm^−1^, which are associated with the bending and stretching modes of O–H groups, respectively [31]. The intensity changes observed after composite formation reflect interactions between Bi_2_MoO_6_ and Ag_2_CrO_4_, indicating the establishment of an intimate heterojunction interface.

### 3.5. Optical Absorption Properties

**Figure 6:**
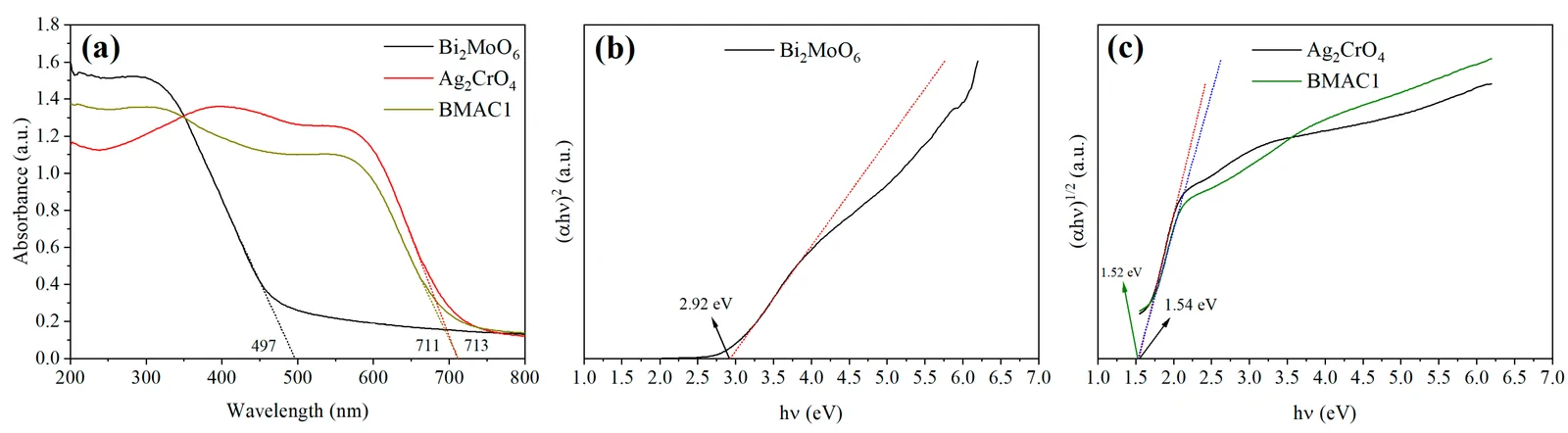
Diffuse reflectance spectra (**a**) together with the Tauc plots (**b**,**c**) for Bi_2_MoO_6_, Ag_2_CrO_4_ and BMAC1.

Figure 6a compares the UV–Vis DRS of Bi_2_MoO_6_, Ag_2_CrO_4_ and the BMAC1 composite, providing insight into their light-absorption characteristics. The absorption edge of pristine Bi_2_MoO_6_ was only 497 nm, indicating its limited visible-light response and relatively low efficiency of photogenerated charge carrier separation. The absorption edge of pristine Ag_2_CrO_4_ reached 711 nm, demonstrating its excellent visible-light harvesting capability for the generation of electron–hole pairs, although it is susceptible to photocorrosion. The BMAC1 composite exhibited an absorption edge of 713 nm, which is significantly longer than that of pristine Bi_2_MoO_6_, indicating the substantially enhanced visible-light response. Therefore, the in situ constructed heterojunction not only induced a distinct red shift in the optical absorption edge of Bi_2_MoO_6_ but also remarkably enhanced its visible-light absorption intensity. These results suggest that more incident photons could be utilized to generate photogenerated electron–hole pairs, thereby effectively suppressing the rapid recombination of charge carriers.

The band gap energies of Bi_2_MoO_6_, Ag_2_CrO_4_ and BMAC1 were further determined, and the results are presented in Figure 6b,c. The band gap energy (E_g_) was calculated according to the Tauc equation [32]:

(3) $${\left(\alpha hv\right)}^{n}=A(hv-E_{g})$$

In the above expression, α corresponds to the absorption coefficient; *h* denotes Planck’s constant; *ν* is the frequency of the incident light, and A is a material-specific parameter. The optical transition of Bi_2_MoO_6_ follows a direct band-gap model (*n* = 2), while Ag_2_CrO_4_ obeys an indirect transition model with *n* = 1/2.

Band-gap values derived from the UV–Vis DRS results are 2.92 eV for Bi_2_MoO_6_, 1.54 eV for Ag_2_CrO_4_ and 1.52 eV for BMAC1. The slight decrease in the band gap after heterojunction formation indicates an extended visible-light response, which enables more efficient absorption of solar energy and promotes the excitation of electron–hole pairs under irradiation.

**Figure 7:**
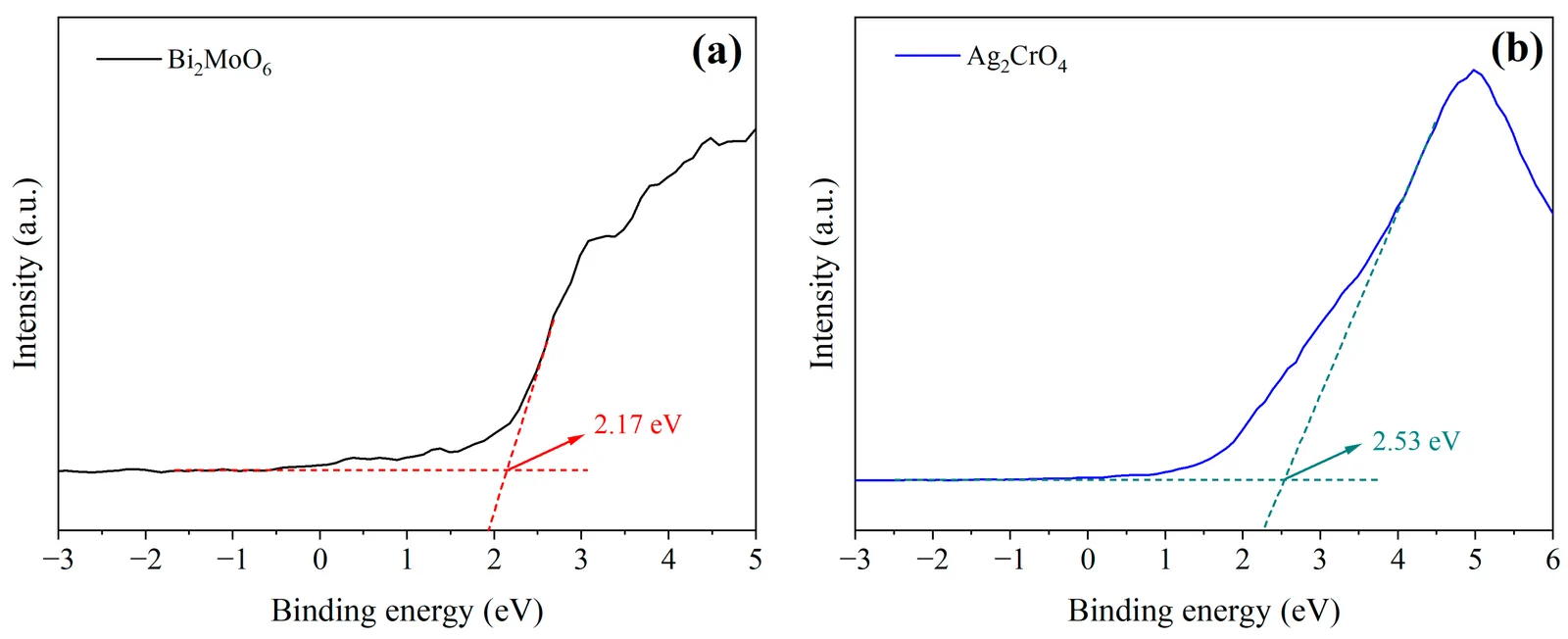
VB-XPS spectra of Bi_2_MoO_6_ (**a**) and Ag_2_CrO_4_ (**b**).

The band edge positions of Bi_2_MoO_6_ and Ag_2_CrO_4_ were determined by VB-XPS, and the results are presented in Figure 7. The valence band potentials (E_VB_) of Bi_2_MoO_6_ and Ag_2_CrO_4_ were determined to be 2.17 and 2.53 eV, respectively. According to the relationship E_CB_ = E_VB_–E_g_, the corresponding conduction band potentials (E_CB_) of Bi_2_MoO_6_ and Ag_2_CrO_4_ were calculated to be −0.75 and 0.99 eV, respectively.

### 3.6. Photoluminescence Analysis

**Figure 8:**
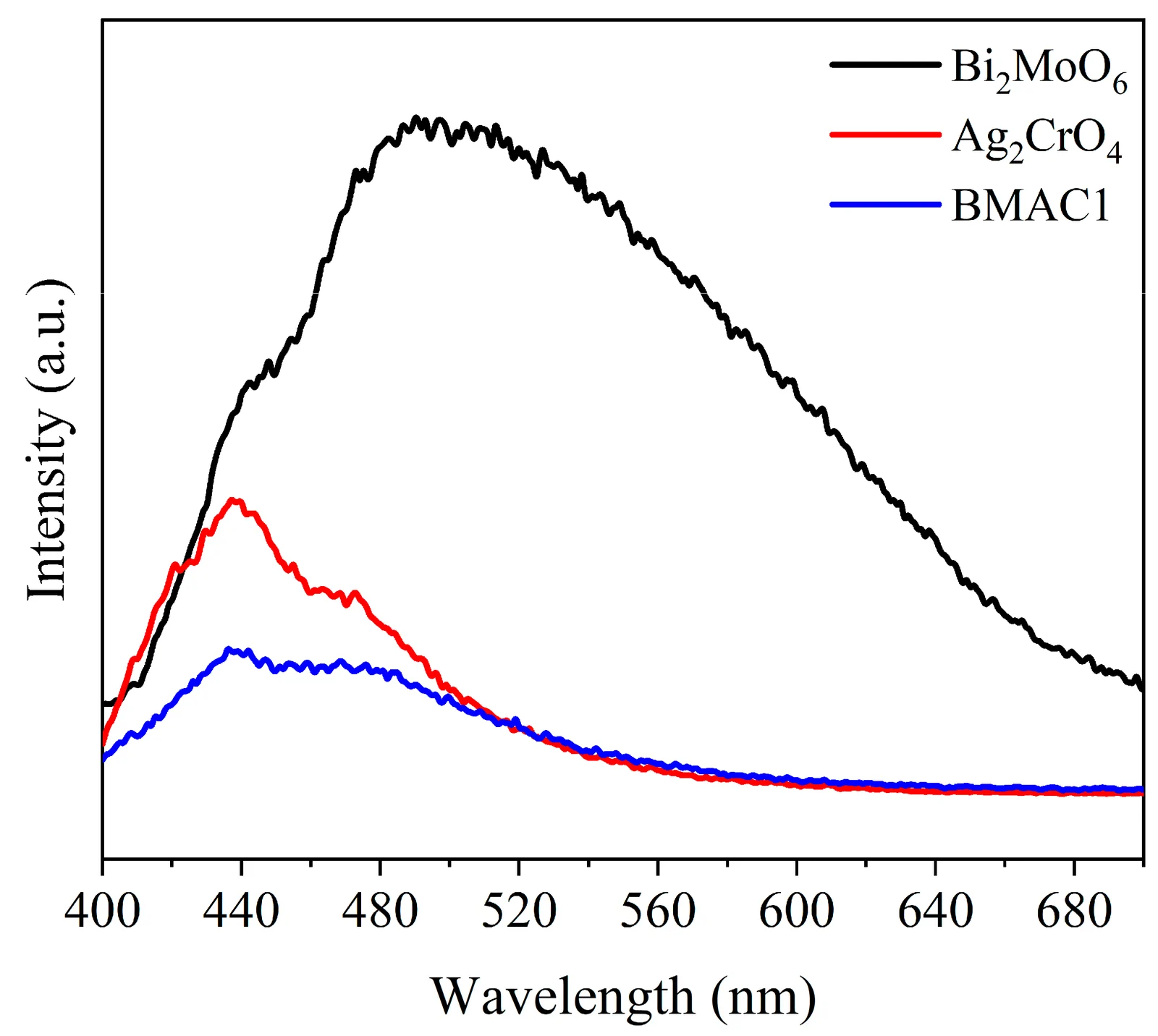
Photoluminescence spectra of Bi_2_MoO_6_, Ag_2_CrO_4_ and BMAC1.

The photoluminescence (PL) spectra of Bi_2_MoO_6_, Ag_2_CrO_4_ and BMAC1 in the wavelength range of 400–700 nm are shown in Figure 8. The PL spectrum of Bi_2_MoO_6_ is characterized by the strongest emission peak among all samples, revealing pronounced recombination of photoexcited carriers. By comparison, the BMAC1 heterojunction exhibits substantially lower PL intensity than either Bi_2_MoO_6_ or Ag_2_CrO_4_, suggesting that the introduction of the heterointerface effectively inhibits carrier recombination. Moreover, BMAC1 exhibited the lowest PL emission intensity in the visible-light region. The pronounced decrease in PL intensity suggests that the recombination of photoinduced electrons and holes is effectively suppressed in BMAC1. Consequently, the heterojunction composite exhibits more efficient charge carrier separation than Bi_2_MoO_6_ and Ag_2_CrO_4_, providing a favorable basis for improved photocatalytic activity.

### 3.7. Photocatalytic Activity and Cycling Stability

**Figure 9:**
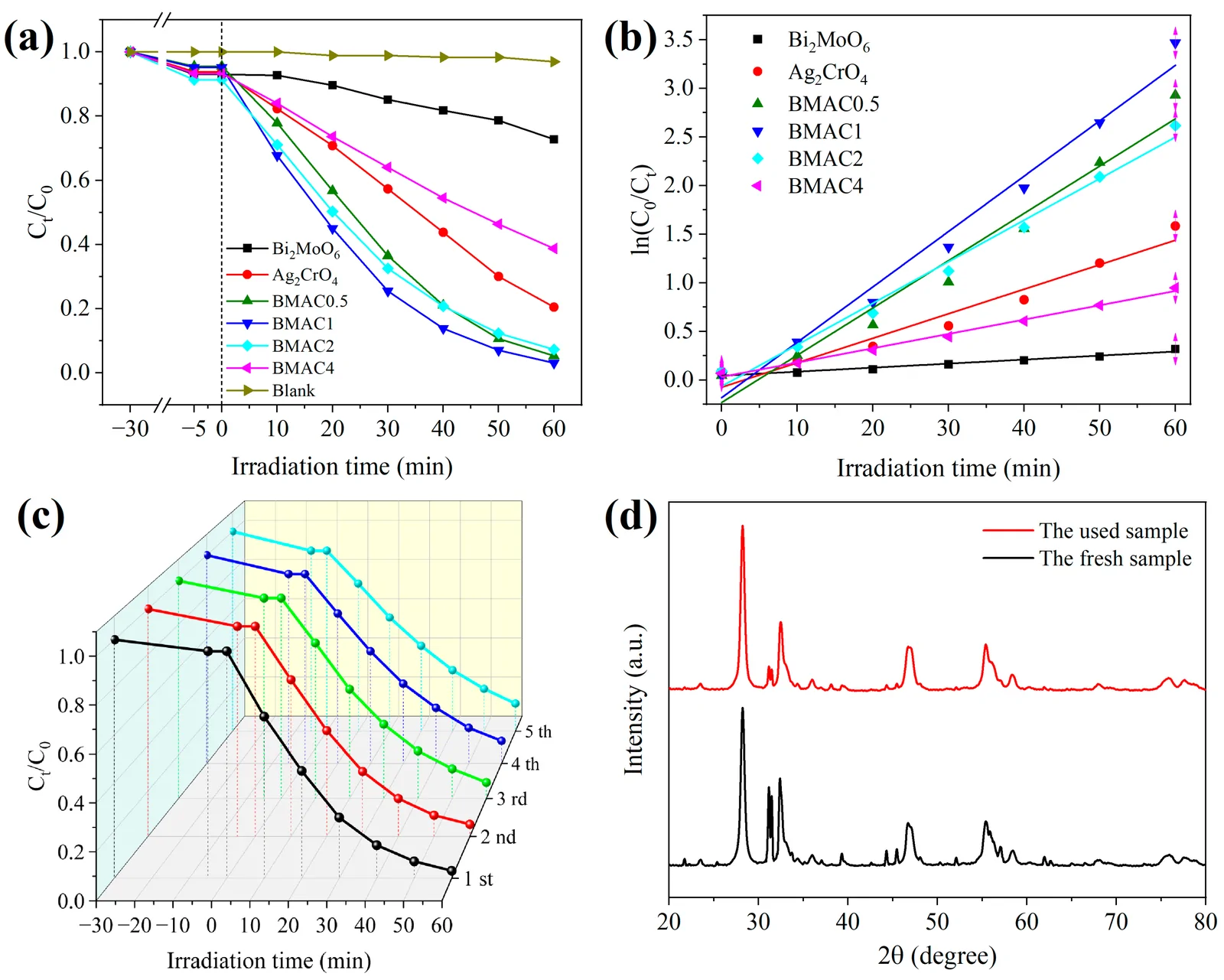
Photocatalytic degradation efficiencies (**a**) and pseudo-first-order kinetic plots (**b**) for RhB degradation over Bi_2_MoO_6_, Ag_2_CrO_4_ and BMAC1; cycling photocatalytic degradation performance of BMAC1 toward RhB (**c**); and phase structure of BMAC1 before and after five consecutive photocatalytic runs (**d**).

The photocatalytic activity and reusability of the Bi_2_MoO_6_/Ag_2_CrO_4_ heterojunction nanocomposites were evaluated by degrading RhB under visible-light illumination, and the results are compiled in Figure 9. As depicted in Figure 9a, in the absence of any catalyst, only 3.07% of RhB was removed within 60 min of light exposure. For the individual components, the dark-adsorption equilibria of Bi_2_MoO_6_ and Ag_2_CrO_4_ toward RhB were determined to be 7.02% and 6.18%, respectively. After 60 min of illumination with visible light, RhB removal reached 27.25% for pristine Bi_2_MoO_6_ and 79.49% for pristine Ag_2_CrO_4_. Upon coupling Bi_2_MoO_6_ with Ag_2_CrO_4_ to construct heterojunctions at various molar ratios, all the composite samples showed a pronounced enhancement in photocatalytic activity. Specifically, the BMAC1 sample exhibited a dark adsorption of 4.82% for RhB, while the RhB removal efficiency reached 96.88% after 60 min of visible-light irradiation. In other words, the photocatalytic activity of BMAC1 was approximately 3.56 times higher than that of pristine Bi_2_MoO_6_. The RhB degradation efficiencies of BMAC0.5, BMAC2 and BMAC4 after 60 min of reaction were 94.66%, 92.70% and 61.24%, respectively. These results clearly indicate that the molar ratio of Bi_2_MoO_6_ to Ag_2_CrO_4_ has a pronounced influence on the photocatalytic activity of heterojunction composites. For instance, photocatalytic degradation performance of BMAC4 toward RhB was even lower than that of pristine Ag_2_CrO_4_. Among all the prepared samples, BMAC1 exhibited the optimum photocatalytic performance.

Figure 9b and Table 1 present the photocatalytic degradation kinetics and the corresponding kinetic parameters for RhB degradation over Bi_2_MoO_6_, Ag_2_CrO_4_ and the Bi_2_MoO_6_/Ag_2_CrO_4_ composites. The degradation of RhB is well described by the pseudo-first-order kinetic model. As the Ag_2_CrO_4_ content increased, the apparent reaction rate constant first increased, reached a maximum at the optimal composition, and then declined with further addition of Ag_2_CrO_4_. After 60 min of visible-light irradiation, BMAC1 exhibited the highest apparent rate constant for RhB degradation, reaching 0.0570 min^−1^, which was 13.90, 2.26, 1.17, 1.33 and 3.88 times those of Bi_2_MoO_6_, Ag_2_CrO_4_, BMAC0.5, BMAC2 and BMAC4, respectively. The outstanding photocatalytic performance of the BMAC1 heterojunction nanocomposite under visible-light irradiation is consistent with the UV–Vis DRS results.

Figure 9c,d illustrate the reusability and cycling stability of the BMAC1 composite photocatalyst. The cycling experiments demonstrate that the photocatalytic activity of BMAC1 decreases only slightly after repeated operation. Following five reuse cycles under 60 min of visible-light irradiation, the RhB removal efficiencies were 96.88%, 94.62%, 92.63%, 89.24% and 86.12%, respectively. Notably, BMAC1 still removed more than 85% of RhB after five successive cycles, demonstrating its excellent reusability. These results indicate that the BMAC1 nanocomposite possesses good photocatalytic durability. Furthermore, XRD analysis was performed on BMAC1 before and after the cycling tests. As depicted in Figure 9d, although the diffraction peak intensities of the recycled photocatalyst decreased slightly, no discernible peak shift was observed, and the major diffraction peaks remained sharp and well preserved. Overall, these results demonstrate that the as-prepared Bi_2_MoO_6_/Ag_2_CrO_4_ heterojunction nanocomposite possesses excellent reusability and structural stability.

### 3.8. Effect of Photocatalyst Dosage

**Figure 10:**
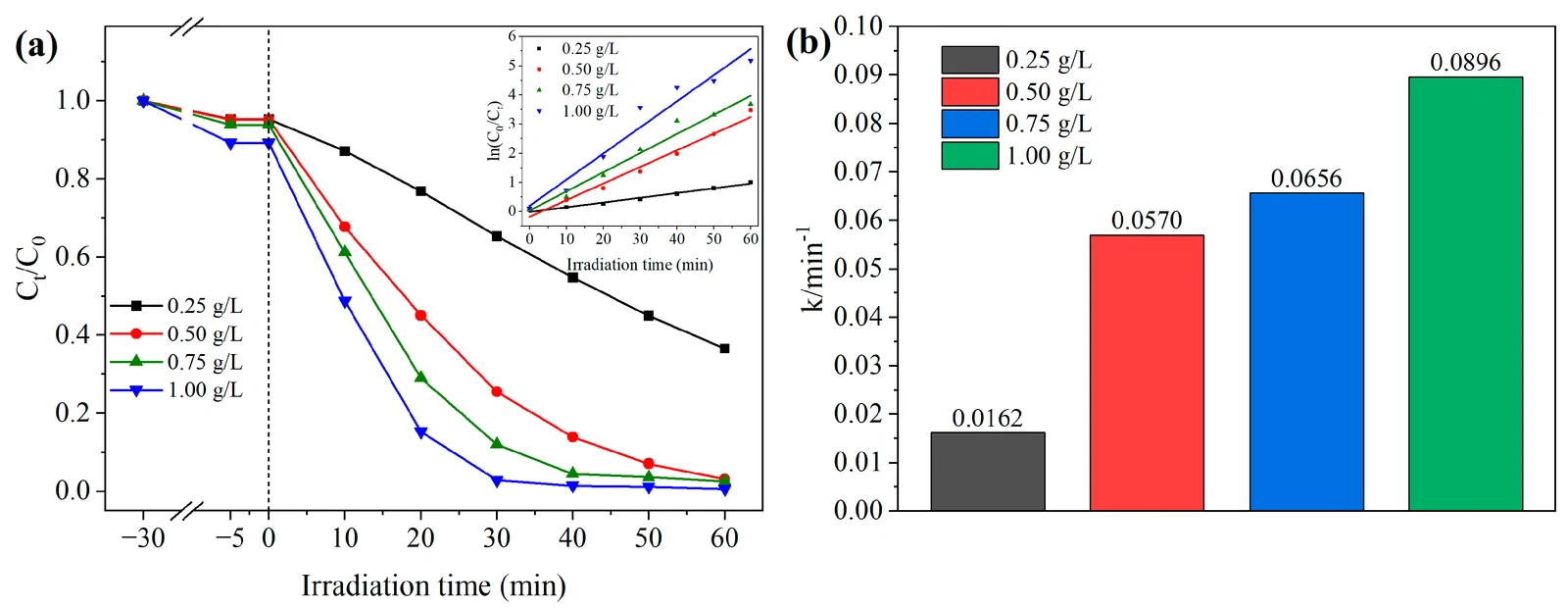
Effect of BMAC1 dosage on RhB degradation efficiency (**a**) and reaction kinetics (**b**). C_RhB_: 10 mg/L.

Figure 10 illustrates the influence of BMAC1 dosage on the photocatalytic degradation of RhB. An increase in catalyst dosage resulted in a progressive improvement in the dark adsorption of RhB by BMAC1. When the dosage was adjusted from 0.25 to 1.00 g/L, the adsorption efficiencies increased from 4.75% to 10.76%. The improved adsorption performance is attributed to the greater surface area and the higher density of adsorption-active sites available at elevated catalyst concentrations. As the photocatalyst dosage increased, the RhB degradation efficiency after 60 min of visible-light irradiation gradually improved. The final RhB removal efficiencies corresponding to BMAC1 dosages of 0.25, 0.50, 0.75 and 1.00 g/L were 63.41%, 96.88%, 97.49% and 99.43%, respectively. Obviously, a higher catalyst dosage could provide more active sites, thereby generating a greater amount of reactive species for the degradation of target pollutant. It is noteworthy that when the BMAC1 dosage increased from 0.50 to 1.00 g/L, the degradation efficiency improved by only 2.55%. Increasing the catalyst dosage further leads to stronger light scattering and shielding within the suspension, resulting in reduced photon availability for photocatalytic reactions on the BMAC1 surface. Therefore, the optimum photocatalyst dosage employed in this study was determined to be 0.50 g/L.

As illustrated by the kinetic fitting curves in the inset of Figure 10a, RhB degradation over BMAC1 can be satisfactorily described using a pseudo-first-order kinetic model. Increasing the catalyst dosage resulted in a progressive increase in the apparent reaction rate (Figure 10b), with rate constants of 0.0162, 0.0570, 0.0656 and 0.0896 min^−1^ for catalyst loadings of 0.25, 0.50, 0.75 and 1.00 g/L, respectively. The improved reaction kinetics are attributed to the higher density of accessible active sites, which enhances interfacial photocatalytic reactions.

### 3.9. Effect of Initial RhB Concentration

**Figure 11:**
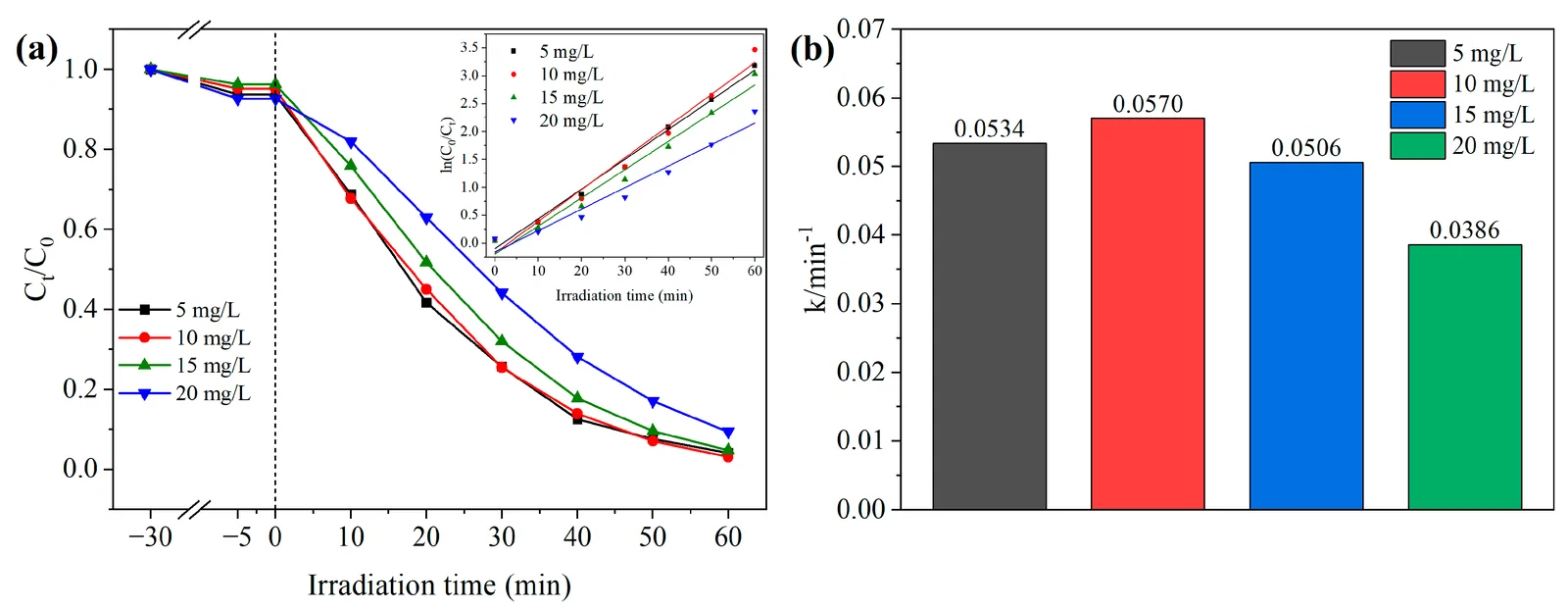
Effect of initial concentration of the RhB solution on RhB degradation efficiency (**a**) and reaction kinetics (**b**). BMAC1 dosage: 0.50 g/L.

The effect of the initial RhB concentration on its photocatalytic degradation was investigated, and the results are presented in Figure 11. With increasing initial RhB concentration, the adsorption efficiency of BMAC1 toward RhB initially decreased and then increased. An adsorption efficiency of 7.35% was obtained when the initial RhB concentration was 20 mg/L. The photocatalytic degradation efficiency initially increased and then decreased with enhancing initial RhB concentration. Variation in the initial RhB concentration from 5 to 20 mg/L resulted in degradation efficiencies of 95.83%, 96.88%, 95.18% and 90.58%, respectively. These results indicate that an initial RhB concentration of 10 mg/L enabled BMAC1 to achieve the highest photocatalytic degradation efficiency. As the pollutant concentration increased to an appropriate level, the probability of contact between RhB molecules and the active sites on the BMAC1 surface increased, thereby promoting the photocatalytic degradation process. However, when the initial RhB concentration exceeded 10 mg/L, the generated reactive species were insufficient to rapidly degrade all RhB molecules. This is because the photocatalyst dosage remained constant, resulting in a nearly fixed amount of reactive species generated within a given period.

Pseudo-first-order kinetic fitting adequately describes the degradation behavior of RhB under different initial concentrations, as shown in the inset of Figure 11a. The degradation rates were calculated to be 0.0534, 0.0570, 0.0506 and 0.0386 min^−1^ for RhB concentrations ranging from 5 to 20 mg/L (Figure 11b). Both the degradation efficiency and kinetic constant reached their highest values at an initial concentration of 10 mg/L before declining at higher concentrations. Based on these results, 10 mg/L was chosen as the standard RhB concentration for subsequent investigations.

### 3.10. Photocatalytic Reaction Mechanism

**Figure 12:**
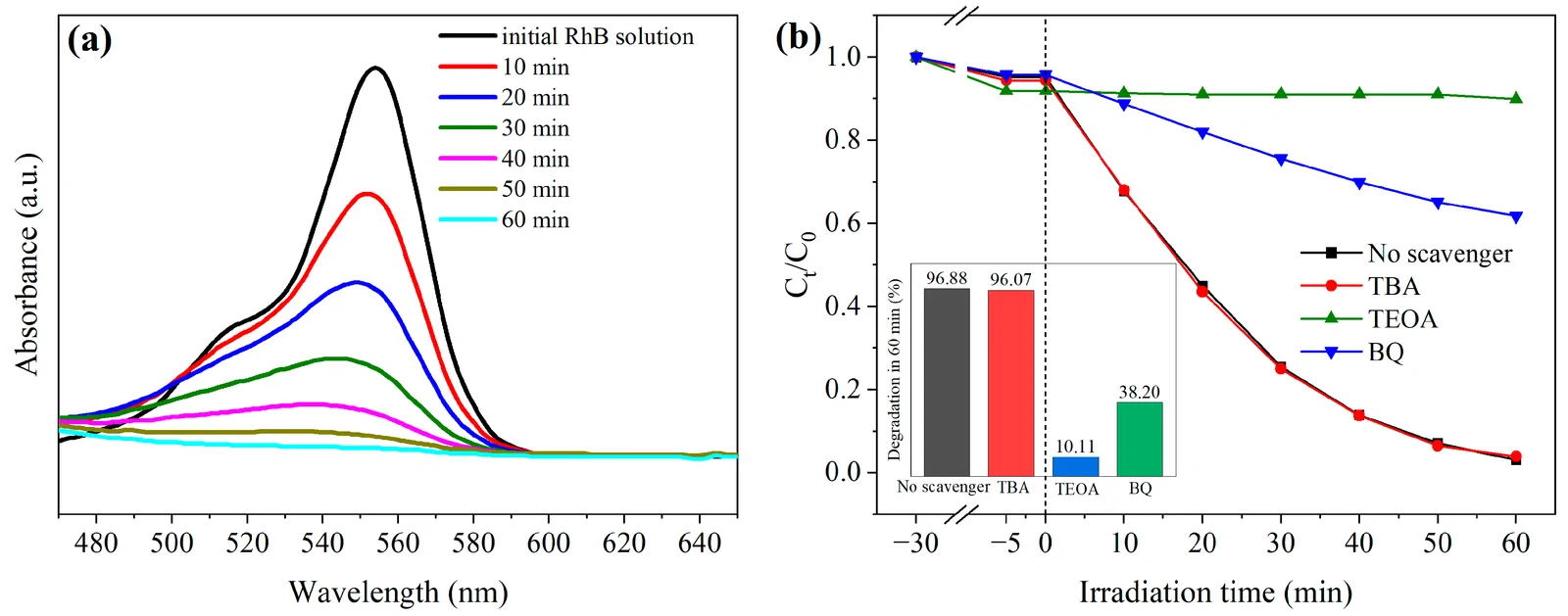
Variation in RhB absorption profiles under visible-light irradiation (**a**) and identification of reactive species through scavenging experiments using BMAC1 (**b**).

The complete degradation of RhB during the photocatalytic process was verified by monitoring its UV–Vis absorption spectra, and the results are presented in Figure 12a. The absorption spectra of the RhB solution were recorded over the wavelength range of 480–640 nm during visible-light irradiation. Monitoring of the UV–Vis spectra revealed a continuous decline in the absorbance of RhB at 554 nm throughout the photocatalytic process. After irradiation for 60 min, the absorption feature nearly disappeared, corresponding to the nearly complete decolorization of the reaction solution. The disappearance of the characteristic absorption peak indicates the complete destruction of the chromophoric xanthene ring structure of RhB molecules, suggesting extensive degradation of the dye molecules [33]. The involvement of these reactive species leads to the cleavage of the conjugated chromophoric system of RhB, thereby promoting its molecular degradation.

A series of quenching experiments was performed with various scavengers to clarify the roles of active species in the BMAC1-mediated degradation of RhB [34]. The RhB degradation efficiency remained nearly unchanged after adding TBA (Figure 12b). After the addition of TBA, RhB degradation efficiency remained as high as 96.07% after 60 min of irradiation, representing a decrease of only 0.81% compared with the control experiment. In contrast, when TEOA and BQ were introduced into the reaction system, the RhB degradation efficiencies decreased dramatically to 10.11% and 38.20%, respectively. The photocatalytic activity of BMAC1 was therefore markedly suppressed. Compared with the system without scavengers, the degradation efficiency decreased by 86.77% and 58.68% in the presence of TEOA and BQ, respectively. The significant inhibition caused by h^+^ and ·O_2_^−^ scavengers confirms that these two species participate actively in RhB degradation. In contrast, the negligible variation observed after ·OH trapping indicates that ·OH makes only a minor contribution to the photocatalytic reaction over Bi_2_MoO_6_/Ag_2_CrO_4_.

**Figure 13:**
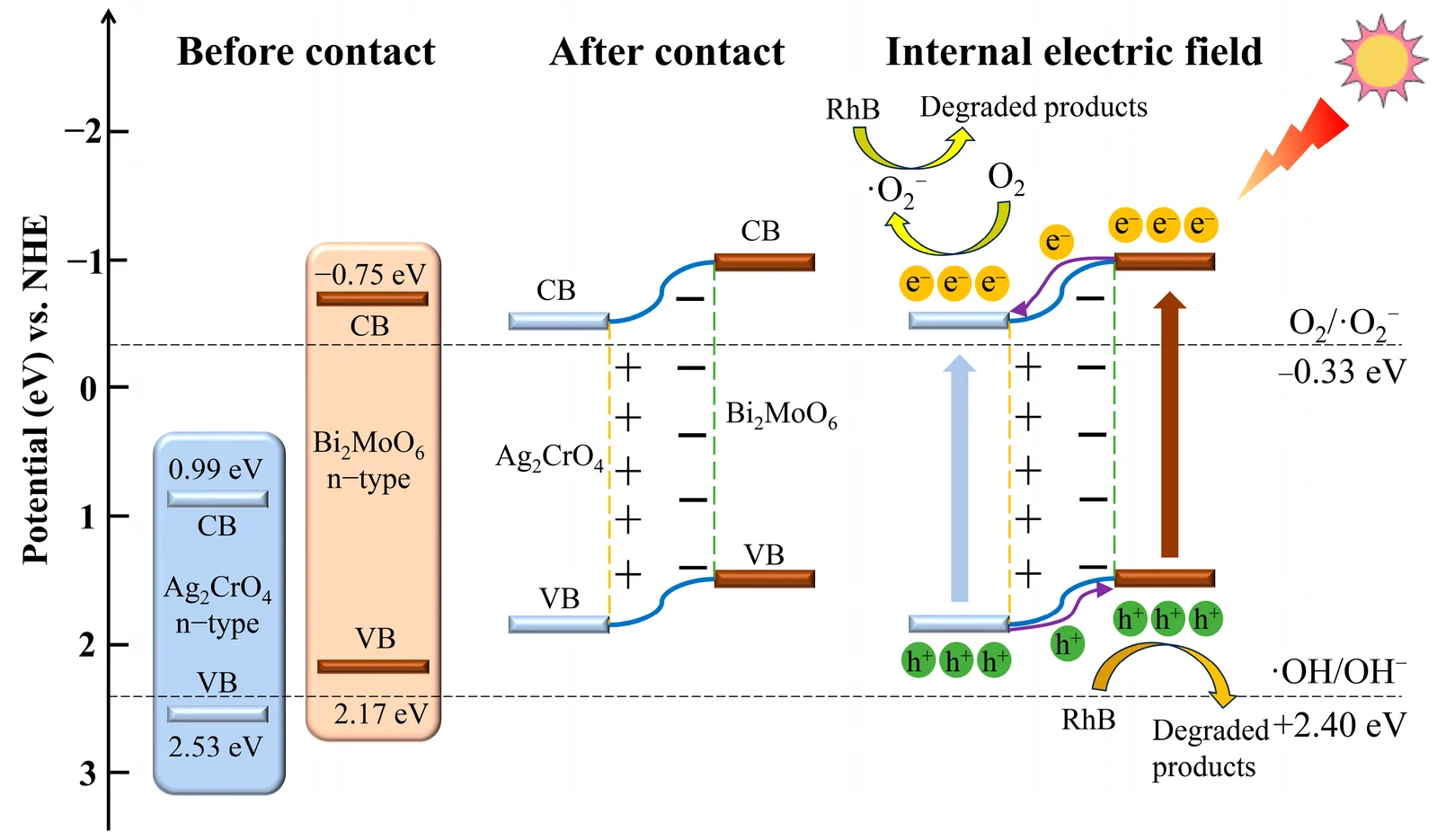
Schematic illustration of the photocatalytic degradation mechanism of RhB on Bi_2_MoO_6_/Ag_2_CrO_4_ under visible-light exposure.

Based on the characterization results, photocatalytic performance analysis, RhB absorption spectra and reactive species trapping experiments, a possible photocatalytic mechanism for RhB degradation over the Bi_2_MoO_6_/Ag_2_CrO_4_ n–n heterojunction was proposed, as displayed in Figure 13. According to the band structure analysis, the VB edges of Bi_2_MoO_6_ and Ag_2_CrO_4_ are positioned at 2.17 and 2.53 eV, respectively, with CB edges of −0.75 and 0.99 eV. Light irradiation excites both components and triggers the generation of charge carriers. In the Bi_2_MoO_6_/Ag_2_CrO_4_ n–n heterostructure, interfacial equilibration between the two semiconductors induces an internal electric field, which regulates the carrier-transfer direction. Consequently, electrons accumulate on Ag_2_CrO_4_, whereas holes are enriched on Bi_2_MoO_6_. The resulting spatial separation of electrons and holes reduces recombination losses and provides more active carriers for photocatalytic reactions.

The photocatalytic degradation reactions are described by Equations (4)–(6). Following interfacial charge migration, the holes accumulated on the VB of Bi_2_MoO_6_ directly participate in the oxidation of RhB molecules. Meanwhile, the electrons enriched on the CB of Ag_2_CrO_4_ are captured by dissolved oxygen to generate ·O_2_^−^. The highly reactive ·O_2_^−^ species further accelerate the oxidative decomposition of RhB, ultimately leading to the formation of CO_2_, H_2_O and other innocuous products. This reaction pathway is consistent with the reactive species trapping results.

(4) $${\mathrm{Bi}}_{2}{\mathrm{MoO}}_{6}{/\mathrm{Ag}}_{2}{\mathrm{CrO}}_{4}+hv\to {\mathrm{Bi}}_{2}{\mathrm{MoO}}_{6}({h_{\mathrm{VB}}}^{+})+{\mathrm{Ag}}_{2}{\mathrm{CrO}}_{4}({e_{\mathrm{CB}}}^{-})$$

(5) $$O_{2}+{\mathrm{Ag}}_{2}{\mathrm{CrO}}_{4}({e_{\mathrm{CB}}}^{-})\to \;•{O_{2}}^{-}$$

(6) $$\mathrm{RhB}+h^{+}/•{O_{2}}^{-}(\mathrm{main})\to {\mathrm{CO}}_{2}+H_{2}O\;\mathrm{or}\;\mathrm{degradation}\;\mathrm{byproducts}$$

## 4. Conclusions

In this study, flower-like Bi_2_MoO_6_ microspheres were successfully synthesized via the solvothermal method, followed by the fabrication of a visible-light-responsive Bi_2_MoO_6_/Ag_2_CrO_4_ n–n heterojunction photocatalyst through an in situ deposition strategy. Structural characterization demonstrated the intimate interfacial contact between Bi_2_MoO_6_ and Ag_2_CrO_4_, with Ag_2_CrO_4_ block-like particles uniformly anchored on the surface of the flower-like Bi_2_MoO_6_ microspheres. The XRD patterns, lattice fringes, XPS binding energies and FT-IR spectra of the composite were all consistent with those of the corresponding pristine components, confirming the successful construction of the Bi_2_MoO_6_/Ag_2_CrO_4_ heterojunction. Compared with pristine Bi_2_MoO_6_, BMAC1 exhibited a substantially extended light absorption edge (713 nm vs. 497 nm), and a significantly lower PL emission intensity, indicating enhanced visible-light harvesting capability and more efficient separation of photogenerated charge carriers. The optimal composite sample (BMAC1) achieved an RhB removal efficiency of 96.88% under visible-light exposure for 60 min. Its photocatalytic activity was 3.56 times that of pure Bi_2_MoO_6_, while its kinetic constant (0.0570 min^−1^) was 13.90 and 2.26 times those of Bi_2_MoO_6_ and Ag_2_CrO_4_, respectively. Moreover, the nanocomposite exhibited excellent reusability and structural stability after repeated cycling tests. The photocatalytic reaction was optimized at a BMAC1 concentration of 0.50 g/L and an RhB concentration of 10 mg/L. The active-species analysis indicated that h^+^ and ·O_2_^−^ dominated the degradation mechanism. The remarkable photocatalytic enhancement of Bi_2_MoO_6_/Ag_2_CrO_4_ was attributed to the improved spatial separation of charge carriers arising from the n–n heterojunction structure, with electrons migrating toward Ag_2_CrO_4_ and holes transferring to Bi_2_MoO_6_. Future research should focus on assessing the risk of metal ion leaching, evaluating the mineralization degree of pollutants, and identifying degradation intermediates to further understand their environmental suitability for practical applications. Overall, this study offers a promising route for constructing efficient visible-light-responsive heterostructured nanocomposites for environmental purification applications.

## Figures and Tables

**Table 1:** Pseudo-first-order kinetic parameters for RhB degradation over Bi_2_MoO_6_, Ag_2_CrO_4_ and Bi_2_MoO_6_/Ag_2_CrO_4_.

Sample	Pseudo-First-Order Kinetic Model Parameters		
	Fitting Equations	Reaction Rate Constant (min^−1^)	R^2^
Bi_2_MoO_6_	y = 0.0446 + 0.0041x	0.0041	0.9494
Ag_2_CrO_4_	y = −0.0743 + 0.0252x	0.0252	0.9510
BMAC0.5	y = −0.2310 + 0.0486x	0.0486	0.9578
BMAC1	y = −0.1812 + 0.0169x	0.0570	0.9774
BMAC2	y = −0.0642 + 0.0226x	0.0427	0.9851
BMAC4	y = 0.0326 + 0.0147x	0.0147	0.9924

## Data Availability

The original contributions presented in this study are included in the article. Further inquiries can be directed to the corresponding authors.
